# Unraveling multilayered extracellular vesicles: Speculation on cause

**DOI:** 10.1002/jev2.12309

**Published:** 2023-02-02

**Authors:** Kelly Broad, Sierra A. Walker, Irina Davidovich, Kenneth Witwer, Yeshayahu Talmon, Joy Wolfram

**Affiliations:** ^1^ Department of Biochemistry and Molecular Biology Department of Physiology and Biomedical Engineering Department of Transplantation Mayo Clinic Jacksonville Florida USA; ^2^ Skaggs Graduate School of Chemical and Biological Sciences University of Florida Scripps Biomedical Research Jupiter Florida USA; ^3^ Department of Chemical Engineering and the Russell Berrie Nanotechnology Institute (RBNI) Technion‐Israel Institute of Technology Haifa Israel; ^4^ Department of Molecular and Comparative Pathobiology The Johns Hopkins University School of Medicine Baltimore Maryland USA; ^5^ Department of Neurology The Johns Hopkins University School of Medicine Baltimore Maryland USA; ^6^ School of Chemical Engineering The University of Queensland Brisbane Queensland Australia; ^7^ Australian Institute for Bioengineering and Nanotechnology The University of Queensland Brisbane Queensland Australia; ^8^ Department of Nanomedicine Houston Methodist Research Institute Houston Texas USA

**Keywords:** ectosome, exosome, microvesicle, multilamellar, unilamellar

## Abstract

Extracellular vesicles (EVs) are cell‐released, heterogenous nanoparticles that play important roles in (patho)physiological processes through intercellular communication. EVs are often depicted as having a single lipid bilayer, but many studies have demonstrated the existence of multilayered EVs. There has been minimal inquiry into differences between unilamellar and multilamellar EVs in terms of biogenesis mechanisms and functional effects. This commentary speculates on potential causes and roles of multilamellar EVs and serves as a call to action for the research community to unravel the complex layers of EVs.

Extracellular vesicles (EVs) are cell‐released nanoparticles involved in intercellular communication and are promising as biomarkers for disease states (Hu et al., 2020; Roy et al., 2018), endogenous therapeutics (Beetler et al., 2022; Iannotta et al., 2021; Roy et al., 2018), and drug delivery platforms (Walker et al., 2019; Witwer & Wolfram, 2021). Cryogenic‐transmission electron microscopy (cryo‐TEM) has allowed the field to assess individual vesicles in a hydrated state, revealing diverse structures that exist within EVs, including multiple lipid bilayers. Multilayered EVs have been identified in conditioned cell culture media and human biofluids, including cerebrospinal fluid, ejaculates, interstitial adipose tissue fluid and plasma (Busatto et al., 2020; Coleman et al., 2012; Emelyanov et al., 2020; Höög & Lötvall, 2015; Issman et al., 2013; Matthies et al., 2020; Poliakov et al., 2009; Tatiana et al., 2020; Tian et al., 2020; Walker et al., 2022; Yang et al., 2022; Yuana et al., 2013; Zabeo et al., 2017). Bias in capturing and selecting images for publishing purposes (Brisson, 2019) is likely to cause an underrepresentation of multilamellar EVs with an overrepresentation of unilamellar (‘conventional’) ones in the literature. There has been minimal investigation into the potential cause and function of multilayered EVs compared to single‐layered ones. It is unclear to what extent multilamellar EVs are formed in native environments as opposed to being artifacts of isolation, storage, and specimen preparation and imaging. This commentary discusses the potential cause and role of multilayered EVs.

Ultracentrifugation, the most common EV isolation method (Gardiner et al., 2016; Konoshenko et al., 2018), has been shown to cause more EV damage than alternative techniques, such as tangential flow filtration and size‐exclusion chromatography (Busatto et al., 2018; Konoshenko et al., 2018; Mol et al., 2017). Therefore, it is worth noting that many studies that reported multilamellar EVs by cryo‐TEM imaging used ultracentrifugation as an isolation method (Coleman et al., 2012; Emelyanov et al., 2020; Matthies et al., 2020; Poliakov et al., 2009; Tatiana et al., 2020; Zabeo et al., 2017). Issman et al. noticed a significant decrease in frequency of multilayered EVs when other methods, such as dead‐end filtration and dialysis, were used instead of high‐speed centrifugation (18,000 × *g*), suggesting that smaller EVs may be forced into larger ones during the centrifugation process (Issman et al., 2013). On the contrary, Matthies et al. demonstrated that multilamellar EVs from conditioned cell culture medium were present to a similar extent with dead‐end filtration and ultracentrifugation (Matthies et al., 2020), both of which can result in membrane damage (Lobb et al., 2015; Shirejini & Inci, 2021; Staubach et al., 2021). However, others have shown that ultracentrifugation primarily causes EV aggregation without the formation of multilayered structures (Linares et al., 2015). A potential reason for this discrepancy is the use of different EV sources (conditioned medium from rat neurons (Matthies et al., 2020) versus human plasma (Linares et al., 2015)) that are likely to have distinct lipid bilayer properties, such as rigidity, which impact the ability of isolation methods to cause multivesicular structures. It has also been shown that tangential flow filtration, which causes less damage than ultracentrifugation and dead‐end filtration (Busatto et al., 2018; Shirejini & Inci, 2021; Staubach et al., 2021), leads to the isolation of both unilamellar and multilamellar EVs from conditioned cell culture medium and biofluids (Busatto et al., 2020; Tian et al., 2020; Walker et al., 2022; Yang et al., 2022). Additionally, a study has noted the presence of multilayered EVs in crude biofluids that were not subjected to isolation protocols (Höög & Lötvall, 2015). In summary, multilayered EVs have been documented with several different isolation methods and in non‐processed samples, suggesting that although some isolation techniques may cause an enrichment in layers (Issman et al., 2013), isolation artifacts are not the sole cause of multilamellar structures.

It is important to note that some studies that reported multilayered EVs, stored samples in −80°C in phosphate buffered saline (PBS) (Emelyanov et al., 2020; Tatiana et al., 2020). There is evidence that −80°C storage of EVs in PBS leads to reduced biological function, decreased concentration, fusion and fragmentation (Bosch et al., 2016; Frank, 2007; Gelibter et al., 2022; Jeyaram & Jay, 2017; Walker et al., 2022). Such storage may also cause membrane damage leading to the formation of multilamellar structures. In particular, freezing and thawing can cause changes in osmotic pressure and formation of ice crystals, which can puncture/weaken membranes, detach biomolecules, and induce conformational changes in membrane proteins (Maroto et al., 2017; Nardid et al., 1997; Qin et al., 2020; Walker et al., 2022). Suggested alternatives to PBS include disaccharides, which have glass transition temperatures that allow protection at −80°C (Bosch et al., 2016; Frank, 2007; Jeyaram & Jay, 2017; Tessier et al., 2021; Walker et al., 2022). Four studies demonstrated the presence of multilamellar EVs after storage at −80°C in a cryoprotective sucrose buffer (Busatto et al., 2020; Tian et al., 2020; Walker et al., 2022; Yang et al., 2022). Other studies reported multilamellar EVs in samples that did not undergo storage prior to cryo‐TEM (Issman et al., 2013; Yuana et al., 2013). Since multilayered EVs were observed with cryo‐TEM in fresh samples (Issman et al., 2013; Yuana et al., 2013) and samples stored in a cryoprotective buffer (Busatto et al., 2020; Tian et al., 2020; Walker et al., 2022; Yang et al., 2022), it is less likely that the presence of multilayered EVs can be solely attributed to storage artifacts.

Taken together, it is unlikely that isolation and storage artifacts are the sole causes of multilamellar EVs. Therefore, it is plausible that various biogenesis mechanisms result in multilayered EVs (Figure 1). For example, it can be speculated that multivesicular structures are formed inside multivesicular bodies through encapsulation of smaller intraluminal vesicles in larger ones, although evidence of this process has not been reported. An alternative mechanism for the formation of multivesicular structures was proposed for lamellar bodies, which are secretory multilamellar organelles found in certain epithelial cells. The proposed model involves flipping of phospholipids from the outer membrane leaflet to the inner one, causing the formation of perpendicular internal membrane sheets that detach, grow, and eventually form curved arrangements (Klein et al., 2021).

**FIGURE 1:**
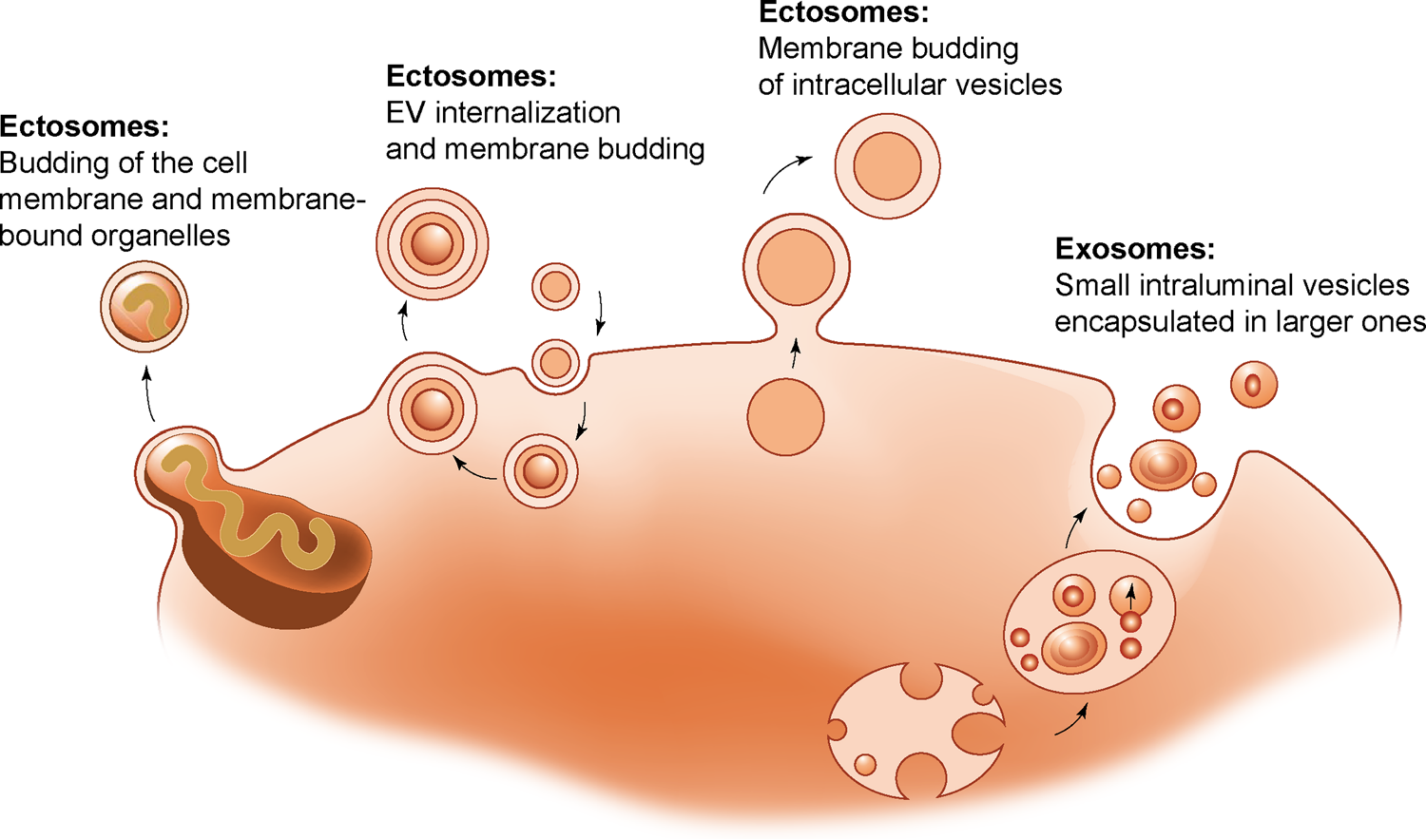
Speculative mechanisms for the formation of multilamellar extracellular vesicles (EVs). An additional speculative mechanism, which has been proposed for lamellar bodies (secretory multilamellar organelles) is illustrated and described in a previous study (Klein et al., 2021).

It can also be speculated that the presence of intracellular vesicles in close vicinity to the cell membrane could result in double‐layer EVs upon membrane budding. In certain cases, intracellular vesicles have been found to encapsulate entire membrane‐bound organelles, such as mitochondria (Phinney et al., 2015). Such mitochondria‐containing vesicles can be transported to the cell surface where outward membrane budding occurs (Phinney et al., 2015). It is also possible that additional EV layers could form through several rounds of cellular internalization, structural preservation, and release through membrane budding, although evidence of this is lacking. Gram‐negative bacteria have been shown to release double‐layer EVs through simultaneous budding of the cytoplasmic membrane and outer membrane ( Perez‐Cruz et al., 2013; Toyofuku et al., 2019). This type of EV formation is restricted to prokaryotes with two membranes, although it is possible that eukaryotic membrane‐bound organelles in close proximity to the cell surface could undergo simultaneous budding (fragmentation) with the cell membrane.

While differences between unilamellar and multilamellar EVs in terms of functional effects have not been explored, liposomes and other synthetic particles are frequently designed to have multiple layers to enable controlled and sustained drug delivery (Boyer & Zasadzinski, 2007; Chen et al., 2020; Peyret et al., 2017; Scavo et al., 2015; Seong et al., 2018; Shen, 2015). In such cases, cargo release is obtained through stimulus‐sensitive or passive degradation‐mediated removal of layers. Multilamellar liposomes also enable increased loading of hydrophobic drugs that are embedded in the bilayers (Kulkarni & Vargha‐Butler, 1995). Therefore, if form follows function, it is reasonable to speculate that EVs have similar mechanisms for controlled/sustained delivery of cargo and increased packaging ability of membrane‐embedded components.

Studies also suggest that the formation of multilamellar EVs is impacted by pathological processes and disease states. For example, Tatiana et al. noted that more multilayered EVs were found in the plasma of Gaucher disease patients (Tatiana et al., 2020). Gaucher disease is characterized by an inability to efficiently metabolize glycolipids (Tatiana et al., 2020), which could potentially impact EV biogenesis and degradation or trigger a compensation mechanism consisting of increased intercellular lipid transfer. On the contrary, it was shown that prion‐infected cells have reduced abundance of multilayered EVs, potentially suggesting that prions packaged within EVs interfere with a multilamellar biogenesis process (Coleman et al., 2012).

Studies have also assessed EV morphology upon cell exposure to lipopolysaccharide (LPS), a bacterial endotoxin. In response to this endotoxin, immune cells release EVs with bioactive cargo that initiate inflammatory responses in recipient cells (Gebraad et al., 2018; Puhm et al., 2019; Tang et al., 2016; Wang et al., 2011). Figure 2 and a recent study demonstrate that LPS stimulation of monocytes increases the formation of multilayered EVs (Yang et al., 2022). Additionally, EVs from LPS‐stimulated monocytes were enriched in membrane‐bound glucose transporter‐1 (GLUT1) (Yang et al., 2022), potentially enabled by the increase in multilamellar structures. GLUT‐1 is known to accelerate inflammatory pathways (Peiró et al., 2016), providing a mechanism by which EVs can induce an intercellular signaling cascade triggered by LPS. The presence of multiple layers may also cause slower release of EV cargo in recipient cells, increasing the time period for intercellular communication about threatening signals in the environment. The number of layers could potentially confer time‐dependent information about signals in the intra‐ and extracellular environment. For example, longer exposure to a stimulating agent, such as LPS, may increase EV layers. Another unanswered question regarding multilamellar EVs is whether each internal compartment separated by a lipid bilayer differs in cargo composition.

**FIGURE 2:**
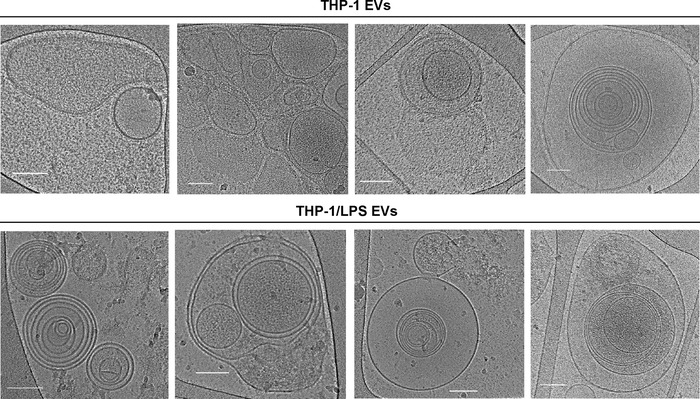
Cryogenic transmission electron microscopy images of EVs from human THP‐1 monocyte‐like cells grown in the absence or presence of lipopolysaccharide (LPS). Scale bars correspond to 100 nm. These specific images have not previously been published, but the methods for cell culture, EV isolation, and imaging are described in a previous study (Yang et al., 2022).

Alternatively, multilamellar EVs may be a mechanism by which to provide recipient cells with large quantities of lipid biomolecules to aid in the repair of potential pathogen‐induced cell damage. It is important to note that LPS may directly cause membrane damage, as studies have shown that this agent can create holes in lipid bilayers (Adams et al., 2014, 2015). Additionally, LPS‐stimulated monocyte‐derived EVs appeared more damaged than those from non‐stimulated conditions (Yang et al., 2022). However, it is unclear whether potential LPS‐induced membrane damage would accelerate EV fusion and/or formation of multilamellar structures. Taken together, metabolic disorders (Gaucher disease) and the presence of prions or bacterial endotoxins have been shown to impact the number of layers present in EVs. The significance of such findings in health and disease remains unknown.

This commentary highlights the lack of research into the cause and role of multilayered EVs, which have been identified in conditioned cell culture media and human biofluids. Further studies are required to determine biogenesis mechanisms, biological function, and relevance of multilayered EVs, which is likely to open opportunities for new treatment strategies, therapeutic targets, and/or biomarkers for diseases, such as those caused by inflammatory signals or metabolic disfunction.

## CONFLICT OF INTEREST STATEMENT

The authors declare no conflicts of interest.
